# Multifunctional Polymeric Nanogels for Biomedical Applications

**DOI:** 10.3390/gels7040228

**Published:** 2021-11-23

**Authors:** Tisana Kaewruethai, Chavee Laomeephol, Yue Pan, Jittima Amie Luckanagul

**Affiliations:** 1 Department of Pharmaceutics and Industrial Pharmacy, Faculty of Pharmaceutical Sciences, Chulalongkorn University, Phayathai Road, Bangkok 10330, Thailand; 6076126733@student.chula.ac.th (T.K.); papomchavee@gmail.com (C.L.); 2 Department of Biochemistry and Microbiology, Faculty of Pharmaceutical Sciences, Chulalongkorn University, Phayathai Road, Bangkok 10330, Thailand; 3 Biomaterial Engineering for Medical and Health Research Unit, Chulalongkorn University, Phayathai Road, Bangkok 10330, Thailand; 4 Guangdong Provincial Key Laboratory of Malignant Tumor Epigenetics and Gene Regulation, Medical Research Center, Sun Yat-Sen Memorial Hospital, Sun Yat-Sen University, Guangzhou 510120, China; panyue@mail.sysu.edu.cn

**Keywords:** polymeric nanogels, stimuli-responsive, functionalized polymer, core-shell nanogels

## Abstract

Currently, research in nanoparticles as a drug delivery system has broadened to include their use as a delivery system for bioactive substances and a diagnostic or theranostic system. Nanogels, nanoparticles containing a high amount of water, have gained attention due to their advantages of colloidal stability, core-shell structure, and adjustable structural components. These advantages provide the potential to design and fabricate multifunctional nanosystems for various biomedical applications. Modified or functionalized polymers and some metals are components that markedly enhance the features of the nanogels, such as tunable amphiphilicity, biocompatibility, stimuli-responsiveness, or sensing moieties, leading to specificity, stability, and tracking abilities. Here, we review the diverse designs of core-shell structure nanogels along with studies on the fabrication and demonstration of the responsiveness of nanogels to different stimuli, temperature, pH, reductive environment, or radiation. Furthermore, additional biomedical applications are presented to illustrate the versatility of the nanogels.

## 1. Introduction

Drug delivery systems (DDSs are platforms that protect loaded active ingredients from degradation due to physiological conditions, as well as allowing the payloads to perform their desired activities at the target sites. Furthermore, some chemicals and biological drugs are cytotoxic to some extent, which is referred to as an undesired off-target effect. DDSs, such as inorganic nanotubes [1], liposomes [2], and inorganic [3] or polymeric nanoparticles [4], have been developed to overcome these disadvantages. A polymeric nanogel is classified as containing small nanoparticles (ca. 20–200 nm) that is fabricated using hydrophilic polymers as the main component. The crosslinked networks of the polymeric chains encapsulate and enhance the colloidal stability of the loaded molecules, and the hydrophilicity of the polymers entraps large amounts of water, which facilitate the diffusion and mass-exchange with physiological milieu, resulting in a controlled or sustained release of the payload.

Most drugs exhibit low solubility and stability; polymer modification, i.e., a smart polymer, has been used to overcome these limitations. Smart polymers refer to a modification or functionalization of the polymers with side chains or ligands to adjust the polymer hydrophobicity [5,6,7]. Additionally, smart polymers can be designed to display distinct stimuli-responsive behaviors, e.g., thermo-, pH-, and redox-responsiveness [8,9,10,11,12,13,14]. These environmentally sensitive features have been extensively investigated because site-specific interactions of the DDS can increase specificity, targeted drug release [15,16,17,18], and diminish side effects [19,20]. Moreover, the improved properties of smart nanogels can be used as diagnostic or theranostic devices [21,22,23,24]. Smart polymers can be designed to specifically respond to various stimuli, light, radiation, an electric field, or temperature, which could be advantageous for target-specific applications [25,26,27]. Therefore, smart polymeric nanogel systems are promising drug delivery platforms with specificity and safety that can be used in a wide range of biomedical applications, such as an effective therapy or an accurate diagnostic system.

## 2. Core-Shell Structure of Polymeric Nanogels

The core-shell structure of nanogels is composed of inner and outer layers that contribute to the functionality of the delivery system. The properties of the core or the inner compartment can be adjusted to protect a loaded substance from incompatible environments or provide a hydrophilic cavity for the hydrophilic therapeutic substances. Nanogels can be fabricated using two or more distinct polymers, and thus the ratio of hydrophilicity and hydrophobicity can be adjusted to meet the requirements of various types of substances. Moreover, the amphiphilicity of the nanogel can transport substances with a low aqueous solubility through the circulatory system. Thus, the inner layer is typically fabricated to enhance the capability of the nanogel to hold and/or stabilize the payload, while the outer compartment, the so-called shell, covers the core, and acts as a protective layer exposed to the surrounding environment. Furthermore, the shell can be modified with specialized functional groups for specific features, such as targetability, stimuli-responsiveness, colloidal stability, or increased retention time in the circulatory system.

**Figure 1:**
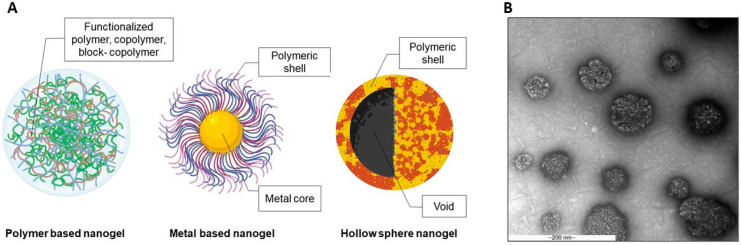
(**A**) Core-shell structures of nanogels (**B**) TEM image of hyaluronic acid-based polymeric nanogels (unpublished data).

In 2011, He F. et al. [28] developed multi-responsive semi-interpenetrating network (semi-IPN) hydrogels whose nanostructure comprised core-shell spherical nanoparticles. The stimuli-responsive semi-IPN hydrogels were modified by magnetic nanoparticles with Fe_3_O_4_ nanoparticles as a core structure together with the combination of poly-N-isopropylacrylamide (PNIPAM, thermo-responsive) and polyacrylic acid (PAA, pH-responsive). This study demonstrated the successful fabrication of multifunctional materials using core-shell structure nanoparticles [28]. Gonzalez-Urias A. et al. [29] introduced pH-sensitive core-shell nanoparticles using a poly (N,N-diethylaminoethyl methacrylate) (PDEAEMA; anionic polymer) or poly (2-methacryloyloxi benzoic acid) (P2MBA; cationic polymer) core shielded with polyethylene glycol (PEG) that can be used for treating cancer. These nanogels can specifically deliver cisplatin, a cancer drug, to a tumor by targeting its acidic environment. The results indicated that the charge of the inner layer influenced nanogel drug release, cell viability, and cell internalization. Additionally, core-shell nanogels composed of gold nanoparticles as a core and biodegradable chitosan as the outer segment were developed to enhance curcumin cytotoxicity to cancer cells [30]. The findings demonstrated an improved cell uptake of the nanogels in breast cancer cells, which resulted in increased toxicity to the cancer cells. This study revealed that polymer and metal materials could be combined to form core-shell nanoparticles for cancer therapy. The findings of these studies indicate that the core-shell structured nanoparticles can be developed from a wide variety of materials to modify the materials with characteristics specific to the required therapeutic properties of the nanogel. In this review, the nanogels systems were categorized into two groups based on the major components of the core or shell of the nanoparticles. Additionally, hollow sphere drug delivery systems with a functionalized shell and empty core are discussed. Illustrations of the three types of nanogels are presented in Figure 1A.

The homogeneity of the nanogel is critical for the stability of the system, and thus the nanogel macrostructure was frequently investigated using transmission electron microscope (TEM). Figure 1B illustrates hyaluronic acid-based nanogel’s TEM images. Likewise, nanogel microstructure was also observed after freeze drying that demonstrated the polymer’s porous structure and interconnected network [31]. The porosity of the microscale structure can increase the mass encapsulation of specific substances, such as oxygen or nutrients [32]. Moreover, the void space between the polymer network increases the diffusion of the nanogels compared with hydrogels [33]. 

### 2.1. Polymer Based Polymeric Nanogels

A nanogel is a colloidal system consisting of a crosslinked, water-swellable, 3-dimensional polymeric network whose size can reach ~1000 nm in a fully-swelled state [34]. The crosslinked network of a nanogel can swell or shrink based on external physical or chemical stimuli. This morphological reversibility leads to the use of nanogels as a smart drug carrier because the site-specific drug release is controllable. Furthermore, several materials possess unique characteristics that are beneficial for the functional design of nanogels. Hydrophobically modified polymers are used to cross the lipid bilayer structure of the cell membrane for an effective cell-targeted delivery system. Liechty WB. et al. [35] introduced the tunability of the hydrophobicity and polymer charge by incorporating hydrophobic moieties, tert-butyl methacrylate (TBMA) and 2-(tert-butylamino)ethyl methacrylate (TBAEMA), to the pH-responsive polymer, P(DEAEMA-g-PEGMA; PDET) [36]. 

The presence of cationic moieties can destabilize the integrity of the cell membrane, which possesses a negative charge surface, leading to increased cell internalization. In addition, amphiphilic polymers were shown to be internalized by fungal cells in Horvat S. et al. [37]. Thiol-functionalized poly (glycidol) nanogels were fabricated, and the amount of alkyl chain conjugated with the thiol groups was varied to optimize the amphiphilicity of the polymers. These nanogels demonstrated an enhanced antifungal effect of entrapped amphotericin B by lowering the minimal inhibitory concentration or MIC, and reduced biofilm formation after treatment. 

A polymeric nanogel was used as a DDS for gene delivery. Costa D. et al. used a conjugated polyamine (spermine, protamine sulfate, or polyethylenimine) on an ethylene glycol diglycidyl ether (EGDE) backbone to encapsulate plasmid DNA. The polymeric nanogels facilitated the delivery of plasmid DNA and anti-cancer drugs, e.g., doxorubicin, epirubicin, and paclitaxel, by increasing the drug-loading capacity because the active substance chemically bonded with EGDE. Moreover, the EGDE crosslinking is broken by ultraviolet light, resulting in drug release [38].

A hollow sphere, a unique structure that contains an empty space surrounded by a polymer has also been reported as a smart delivery system, because the core can facilitate drug encapsulation and controlled release [39]. Together with the functionalized polymeric shell, hollow sphere nanogels can be site specific or stimuli-responsive. Many research groups have used the distinctive properties of a hollow sphere in biomedical applications because the inner void provides a large space for drug encapsulation and the shell thickness is tunable [40]. 

A combination of thermo-responsive polymers that were chosen to increase the advantages of the hollow sphere structure was described by Li G. et al. The thermo-sensitive polymeric hollow spheres were assembled using sodium alginate-graft-poly(N-isopropylacrylamide) (ALG-g-PNIPAM) and β-cyclodextrin (β-CD) for 5-fluorouracil (5-FU) controlled release. The nanoparticles in the nanorods and coils were prepared using β-CD/PNIPAM and sodium alginate, respectively. The fabricated hollow sphere nanoparticles were expected to increase 5-FU loading. At temperatures above the lower critical solution temperature (LCST) of PNIPAM or in an acidic environment, loaded 5-FU release was enhanced due to the morphological change of the nanoparticles [39]. Furthermore, the characteristics of the hollow sphere can be optimized by modified shell polymers. 

The fabrication of a hollow shell-shell nanocontainer composed of thermo-responsive PNIPAM and poly(N-isopropylmethacrylamide) or PNIPMAM as an inner and outer shell was reported by Schmid AJ. et al. [39]. An in silico study of the shrink-swell behavior of the inner shell demonstrated a controllable uptake and drug release, while the outer shell facilitated colloidal stability and maintains the hollow sphere’s void. More recently, hollow sphere nanogels were used as a skin hydration and penetration enhancer. Osorio-Blanco ER. et al. fabricated a thermo-responsive hollow sphere nanocapsule using PNIPAM and PNIPMAM as shell polymers, which provided thermo-responsive behavior to the system. The ratio of the polymers was varied to optimize the volume phase transition temperature and size of the nanogels. After being triggered by heat, this nanocarrier demonstrated improved skin penetration because it could move through the stratum corneum to the viable epidermis. Interestingly, the nanocapsule exhibited the penetration of a high molecular weight Atto oxa12 (MW = 835 g/mol) into the viable epidermis, which indicated increased skin penetration compared with DMSO, which was used as the positive control [41]. 

Wang Z. et al. introduced gold hollow/nanoshells conjugated with small interference RNA (siRNA) designed to interfere with the expression of heat shock protein 70 (hsp70) that is produced during photothermal therapy (PTT) of a tumor and is associated with tumor resistance and tumor recurrence. In this study, siRNA was entrapped by the gold hollow/nanoshells that protected the siRNA from enzymatic degradation and effectively delivered it to the cell cytosol. Together with the light absorption and light-heat transformation potential of gold hollow/nanoshells, hsp70 was down-regulated, resulting in increased PTT efficacy [42]. An inorganic substance was also incorporated with hollow sphere nanogels. Han H. et al. used PS-PAA (polystyrene- poly(acrylic acid)) core-shell nanogels as a template for hollow silica nanoparticles. After removing the PS-PAA core, a pH-responsive silica hollow sphere was obtained and demonstrated successful pH-triggered drug release [43]. 

Functionalized polymers have also been fabricated using layer-by-layer techniques, the so-called nanofilms [44]. In these core-shell particles, nanofilms were used as a polymeric shell coated on particles or microspheres to facilitate multifunctionality [45,46], specificity [47], controlled release [48], or cytoprotective effects [49]. The various uses of the functionalized polymers represent their advantages as a smart drug delivery material, especially the tunability of the polymers that influence efficacy, specificity, or stimuli-responsiveness of the fabricated carriers.

### 2.2. Metal Based Polymeric Nanogels

Numerous studies have demonstrated wide applications of metal-based nanomaterials, such as catalysts or nanoconductors. The distinct features of metal-based materials, quantum dots and metallic hybrid nanogels, have resulted in their use in biomedical fields [50]. Several metallic substances are light-responsive or emit fluorescence when in an excited state. Hence, the nanogels loaded or conjugated with these materials are used as diagnostic devices, such as optical nanosensors, contrast imaging, and cellular tracking.

Wu W. et al. developed silver (Ag)/gold (Au) dual-metal nanoparticles coated with a hydrophobic polystyrene (PS) layer as an inner core and shieled with PEG. These bimetallic nanoparticles emitted intense fluorescence, which could be used for cell imaging. Moreover, near-infrared (NIR) light can be absorbed by these nanogels, leading to photothermal conversion that can be beneficial for drug release [51]. Au has photothermal conversion activity that can enhance the effectiveness of anti-cancer therapeutics [50], and the use of other metals were also reported. 

Selenium (Se) was conjugated to sulfhydryl groups as Se-S to create reduction-responsive linkages. The linkages were designed to prepare a doxorubicin (DOX)-loaded superparamagnetic nanogel with reduction and pH-responsiveness for drug release. The release of DOX from the magnetic Se-S nanogels coated with alginate was enhanced in the high glutathione (GSH) tumor environment [52]. 

Nanoclusters embedded in nanogels were reported by Gao X. et al. [53]. Copper, as copper nanoclusters (CuNCs) with cysteine ligands, were incorporated into glycol chitosan to form nanocomposites. The modified nanoparticles were designed to detect physiological Zn^2+^, because the level of Zn^2+^ in the human body impacts several biological mechanisms, such as growth and neurotransmission. These nanogels can be localized due to their increased photoluminescence intensity from the transition between the dispersed and aggregated state of CuNCs@GC in the presence of Zn^2+^. This feature provides an interesting tool for live cell imaging.

Quantum dots or QDs exhibit outstanding properties, such as photo-luminescence with less dye fading compared with traditional dyes. This property makes QDs efficient tools for in vitro and in vivo imaging. Adipic acid dihydrazide-modified QDs were conjugated to the carboxyl groups of hyaluronic acid as an initial nanogel polymer. The obtained nanogels demonstrated an intense fluorescence signal after cell uptake, which was related to the affinity of the modified hyaluronic acid-based nanogels to CD44 receptors on the cell surface [54]. Colloidal semiconductor QDs display a unique physical property where the size of the particle is dependent on the optical properties owing to their electronic state limit [55]. 

In multiple QD studies, a QD-modified core–shell structure was designed to replace metals, which significantly reduced the cytotoxicity of the materials. QDs-chitosan core-shell nanogels as an “OFF”/“ON” biosensor were fabricated as a cancer cell targeted probe. This system was composed of Mn^2+^-doped CdS/ZnS, which was a glutathione sensitive QD core, attached to dopamine as an intracellular simulation moiety and model drug. The complex core was coated with chitosan functionalized folic acid as a targeted ligand for cancer cells and fluorescein isothiocyanate (FITC). The interaction between this QD nanogel and cells that expressed different levels of folic acid was demonstrated along with being nontoxic up to 100 mg/L. [56] 

Cancer has become a worldwide health issue because it exhibits complicated pathologies [57] and can metastasize through the blood to distal parts of the human body [58]. Various therapeutic substances and delivery systems were investigated to overcome the challenges of this disease. To estimate the disease severity and efficacy of cancer treatments, tracking nanogels were designed and evaluated in vitro and in vivo. 

Tan L. et al. reported that ZnO nanoparticles decorated with 2,3-dimethylmaleic anhydride (DMMA) and loaded with DOX exhibited a synergistic cytotoxicity to cancer cells under acidic conditions. The ZnO QD system prolonged the retention time in the blood and an accumulated at the tumor that improved the efficacy of the loaded chemotherapeutics [59]. Notably, in Liu Y. et al., the tumor-suppressor gene p53 was effectively delivered using dextran-QD nanogel to elevate its stability and imaging property. The dextran-QD nanogels were functionalized with polycation PGEA (ethanolamine-functionalized poly (glycidyl methacrylate)), and this nanoparticle demonstrated improved gene transfection efficiency and real-time in vivo fluorescent imaging [60]. 

QDs can also be modified by a stimuli-responsive polymer as described in Gui R. et al. In this study, nanospheres of QDs-embedded mesoporous silica nanoparticles (Q-MS) coated with chitosan-functionalized PNIPAM or PNIPAM-g-CS shell were fabricated. The results suggested the potential of nanospheres as a cell imaging agent in Hep2 cells along with the thermo-responsive behavior of PNIPAM, which can further facilitate the specificity to particular target sites [61].

## 3. Stimuli-Responsive Nanogels

Over the past decade, various smart polymers have been introduced as drug carriers with increased effectiveness, specificity, and fewer side effects. Improving polymers by incorporating proteins or other polymers can enhance specific characteristics and overcome some limitations of the base polymers. Furthermore, polymer modification can increase site specificity or cell internalization that can result in increased therapeutic effects.

Arteche Pujana M. et al. demonstrated a biodegradable crosslinker, genipin, grafted on chitosan to modify the water solubility of a chitosan derivative by minimizing its hydrogen bonding. Compared with the unconjugated chitosan, the modified structure exhibited improved water solubility and was pH-responsive that can be used for pH-triggered drug release [62]. The elevated transport of tenofovir across vaginal epithelium using an increased ratio of the unionized/ionized forms of tenofovir after encapsulated in nanoparticles composed of a combination of poly (lactic-co-glycolic acid) and methacrylic acid was demonstrated by Zhang T. et al. [63] The combination of two or more polymers resulted in improved material properties with more efficient drug entrapment and increased site-specificity.

**Figure 2:**
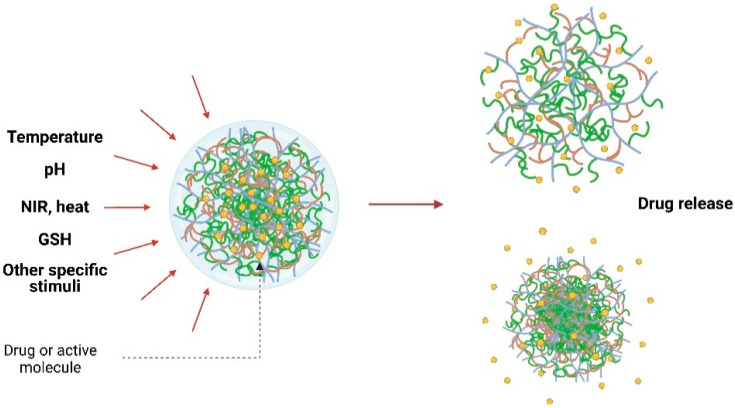
Shrink-swell behavior of nanogels after stimulation by specific stimuli resulting in targeted drug release.

Stimuli-responsive behavior is an important feature for biomedical applications. As presented in Figure 2, various smart polymers respond to diverse stimuli such as pH, temperature, enzymes, light, and chemicals. Several studies illustrated the usefulness of modified polymers where their responsiveness provided improved functions and enhanced delivery system specificity.

### 3.1. Thermo-Responsive Nanogels

Temperature is a stimulus that has been investigated in stimuli-responsive drug carriers because most therapeutic substances have been designed to be activated at physiological temperature. Moreover, some diseases, e.g., cancers, have specific temperatures that can be exploited for targeted therapies. 

Luckanagul J.A. et al. [64] reported the conjugation of a thermo-responsive polymer, PNIPAM, to chitosan that was used as a base material to form a drug carrier to enhance the aqueous solubility of a hydrophobic drug, curcumin. PNIPAM degrades too slowly, therefore conjugation with chitosan improves is biocompatibility due to improved biodegradability and reduced PNIPAM toxicity. This study also investigated the thermo-responsiveness of the modified polymer. The results demonstrated that the hydrodynamic size increased as the temperature increased and the thermo-responsiveness of the nanogels depended on the length and the grafting density. The cytotoxicity of the curcumin-loaded nanogels to cancer cells, MDA-MB-231, Caco-2, HepG2, and HT-29 cells, was evaluated and the results suggested the potential therapeutic effects of the nanogels in several cancer types [64]. 

The thermo-responsiveness of PNIPAM-modified nanogels is finely tunable using β-CD as reported by Yi P. et al. [65]. The result indicated increased thermo-sensitivity of the polymers, which resulted from linking β-CD and the isopropyl group of PNIPAM. The thermo-responsiveness of the nanogel was investigated based on the in vitro DOX release at 25, 37, and 40 °C, and faster release was observed at 40 °C. Moreover, the accelerated release of DOX in acidic pH and high reducing environment was reported, suggesting the use of the nanogels in cancer therapy. 

In addition to PNIPAM, 2-(2-methoxyethoxy)ethyl methacrylate (MEO_2_MA), a thermo-responsive monomer, was incorporated into silver nanoparticles (AgNPs) generating metal-based nanoparticles that were thermo-responsive. Soto-Quintero A. et al. [66] used a one-pot synthesis based on the reducing property of the phenolic moieties of curcumin to induce the formation of MEO_2_MA-conjugated AgNPs and the interfacial energy reduction between MEO_2_MA monomer and the metal surface. The curcumin-incorporated AgNPs exhibited antioxidant and thermo-responsive activities. The metallic core enhanced the capacity to encapsulate the hydrophobic curcumin and provided luminescence that can be beneficial for diagnostic and therapeutic purposes. 

There has been increased attention paid to the diagnostic capability of stimuli-responsive nanogels. Thermo-responsive copolymers for high contrast magnetic resonance imaging (MRI) were initially reported by Kolouchova K. et al. [67]. Two block polymers, poly[N-(2-hydroxypropyl)-methacrylamide] (PHPMA) or poly (2-methyl-2-oxazoline) (PMeOx), and a thermo-responsive poly-[N(2,2difluoroethyl)acrylamide] (PDFEA) were synthesized as amphiphilic copolymers that can undergo self-assembly to form nanogels under physiological conditions. The self-assembled nanogels were used to encapsulate a magnetic-sensitive fluorine to use as high sensitivity MRI nanoparticles.

In addition to drug delivery and diagnostic devices, thermo-responsive polymers can be employed as an initial material in the self-assembly of polymeric nanoparticles. Poly (ethylene glycol)-methacrylate-based maleimide-bearing copolymer was used as the initial nanoaggregate in the reaction of a Clickable-nanogel due to its stability in an aqueous solution. Cell imaging and internalization of the nanogel were evaluated in the study [68].

Thermal triggering can occur by other sources, such as NIR light, which generates heat from the excitation of specific molecules. Moreover, NIR light can penetrate to deep tissues with less damage compared with direct heat or UV light. Shang T. et al. used the core-shell structure of Au_rod_@PNIPAM-PEGMA nanogels (PNIPAM-PEGMA = cross-linking of PNIPAM and poly-(ethylene glycol)-methacrylate (PEGMA)), which responds to NIR light stimulation. Nanoparticles containing the Au rod absorbed the NIR light and converted it to heat. The elevated temperature caused the thermo-sensitive PNIPAM-PEGMA nanogels to shrink and release the payload. Moreover, the molar ratio of PNIPAM to PEGMA was varied and these nanogels demonstrated increased LCST at a reduced molar ratio, revealing the fine tunability of thermo-responsive behavior of the polymer that is useful for its controlled release of a material [69]. 

NIR light-sensitive nanogels with photo-sensing properties were designed by Wang H. et al. as bifunctional nanoparticles composed of a fluorescent carbon dot core coated with PNIPAM-co-acrylamide-based hydrogels. The photo-sensitivity of the carbon dots was used for in vivo imaging of tumor tissue together with the NIR light-triggered temperature increase, resulting in the thermo-responsive behavior of the nanogel for specific drug release [70].

Ma X. et al. [71] reported a nanogel system that was well-designed as a multi-stimuli-responsive carrier of a chemotherapeutic prodrug, 10-hydroxycamplothecin, and a photosensitizer, purpurin18 (P18). The nanogels demonstrated drug release behavior in the reducing tumor microenvironment along with ROS generation because the release of P18 was stimulated using 660-nm laser irradiation. Interestingly, P18 also responded to the NIR excitation, resulting in a real-time monitor of the treatment, and can be further used for diagnosis.

### 3.2. pH-Responsive Nanogels

pH is a physiological factor that affects the ionization state of drug molecules, and influences the behavior of pH-sensitive drug delivery systems. Typically, the pH value at the target sites in several diseases differs from the physiological pH. Thus, designing a drug carrier with a specific pH-responsiveness can enhance the therapeutic efficacy and reduce side effects to the surrounding organs or tissues. Due to the different charge characteristics of each polymer, chemical modification or polymer blending could convey advantages to the distinct functionalities among other nano-carriers. 

The morphology transition of anionic nanogels under different pH conditions was reported by Ashrafizadeh M. et al. [72]. They used a nano-system composed of ionizable acrylic acid, butyl acrylate, and ethylene glycol dimethacrylate as a crosslinker. The modified polymers were pH-responsive, where their swelling was increased at pH 7.5. The morphology of the modified nanogels transformed from a sphere structure at acidic pH to a core-shell structure as the pH increased, before forming a thin-walled sphere as pH further rose (pH >9). The maximum swelling indicated the feasibility of drug release under physiological conditions. Furthermore, Pal S. et al. [73] used a pH-responsive nanogel system composed of dextrin and poly (acrylic acid) using N,N’-methylene bis acrylamide (MBA) as a crosslinker. Protonation of the carboxyl groups of poly (acrylic acid) was responsible for controlling the drug release at an acidic pH (pH 5.5) that can be utilized for cancer therapy. Furthermore, the dextrin provided a greater biocompatibility to human mesenchymal stem cells [73]. 

pH-responsive hyaluronic acid-based nanogels have been fabricated to deliver a terephthalaldehyde (TPA) protein drug, cytochrome C (CC) and enhance cell uptake in the tumor environment [74]. Hyaluronic acid-grafted-methoxy PEG-diethylenetriamine copolymers was crosslinked with TPA by forming a benzoic imine bond. The benzoic imine facilitated the pH-responsiveness of the system because it can convert a negatively charged surface to a positively charged surface in the acidic tumor microenvironment, thereby releasing CC. This study illustrated using a polymeric nano-system to deliver macromolecules that widened the applications of the biologic drug delivery system.

Metallic nanoparticles can be pH-responsive, as demonstrated by Wu W. et al. Ni-Ag nanoparticle core/PEG-co-methacrylic acid shell nanogels were used as a targeted drug carrier for cancer treatment using 5-FU as a model drug. In this study, the metallic core was photoluminescent and its intensity depended on the pH value being 5.0–7.4. The nanogels also exhibited the rapid release of 5-FU at the tumor site due to acidic pH-triggered release. In addition, the drug loading capacity of the hybrid nanogels was high due to the intermolecular complex between the polymeric shell, the ether oxygens of PEG, and carboxyl groups of the methacrylic acid moiety [75].

### 3.3. Reduction-Responsive Nanogels

Tumor cells typically exhibit a higher amount of reducing compounds compared with normal cells. This distinct environment has led to the development of targeted cancer chemotherapy using a drug delivery system that is affected by a reducing environment [76]. GSH-responsive nanogels consisting of a poly (oligo (ethylene oxide) monomethyl ether methacrylate) crosslinked network and disulfide-functionalized dimethacrylate were synthesized in which the disulfide bond could be cleaved by a high GSH concentration. Indeed, a water-soluble fluorescent dye, Rhodamine 6G (R6G), was readily released when the R6G-loaded nanogels were incubated with C2C12 cancer cells, confirming a reduction-sensitive release. DOX, an anticancer drug, was also loaded in the nanogels and an enhanced cytotoxicity to HeLa cells was observed [77]. 

Reduction-responsive nanogels have been extensively reported as intracellular targeting delivery systems, because the intracellular concentration of GSH ranges from 1–10 mM, which is relatively high compared with the extracellular space [78]. This cell environment results in intracellular-specific payload release. Carboxyl-functionalized polyvinylpyrrolidone nanogels were developed with (3-[(2-aminoethyl)dithio]propionic acid), a redox-sensitive spacer that contains disulfide bridges. The highest release of DOX was achieved in the presence of a reductant, dithiothreitol, due to the reductant sensitivity of disulfide bonds that led to the cleavage and relaxed polymeric networks. 

This reduction-triggered release system was also used for siRNA delivery, due to the instability of siRNA in the circulatory system and the low transfection efficiency of bare nucleotides. The siRNA was conjugated with polyvinylpyrrolidone to form nanogels with reduction-sensitive bonding without losing the inherent silencing properties [79]. Furthermore, plasmid DNA can be efficiently delivered using the nanogels as reported by Liwinska W. et al. [80]. Plasmid DNA was crosslinked to the base polymers containing disulfide bonds, thus, the disulfide cleavage occurred in the high GSH intracellular environment. 

The use of metal-containing GSH-responsive nanogels, composed of poly [methacrylic acid-co-poly(ethylene glycol) methyl ether methacrylate, as a tumor-targeted drug carrier was reported [81]. N,N-bis(acryloyl)cystamine facilitated the nanogels to respond to the GSH gradient along the distance from the external part of tumors to the core. Along with the encapsulated DOX, in vitro studies demonstrated markedly reduced release at the tissue periphery based on the drug-leakage protection provided by the crosslinked metal and accelerated drug release at the tumors due to the highly concentrated GSH in the tissue. 

Glucose is also a stimulus that can be used when designing a responsive drug delivery system, especially for diabetes mellitus [82] or infection [83]. Several glucose-sensitive hybrid nanogels were designed to respond to specific glucose levels and specifically release glucose-lowering agents. Phenylboronic acid is a polymer used for sensing glucose levels as a phenylborate ion or the dissociated (charged) form that can react with the 1,2-cis-diols of glucose in an aqueous solution [84]. Poly (4-vinylphenylboronic acid-co-2-(dimethylamino)ethylacrylate), a boronic acid derivative, was coated on an Ag core to form nanogels that are glucose-sensitive. The glucose binding by nanogels was investigated based on the swelling of the particles in higher glucose concentrations, enhancing the release of entrapped molecules.

Infected cells exhibit a high level of mannose receptors on their surface, and thus mannosyl ligand-functionalized nanogels were fabricated target infected tissues. The nanogels were then ingested by macrophages at the infection site. Additionally, the bacteria at the infected site can secrete vesicles containing phosphatase and/or phospholipase, which disassociated the PEG grafted-polyphosphoester. Nanogels made of this material showed an accelerated release of loaded vancomycin and cytotoxicity to methicillin-resistance *Staphylococcus aureus* (MRSA).

### 3.4. Multi-Responsive Nanogels

To provide additional functionality to nanogels, multi-responsive features, which are responsive to two or more stimuli, were introduced to deliver drugs with higher efficacy and safety. Temperature-, pH-, and NaCl-sensitive nanogels were demonstrated by Xu Y. et al. to be dual-function injectable nanocarriers that enhanced drug delivery and functioned as biological probes [85]. This multi-responsive delivery system, composed of PNIPAM-co-adenine (PNIPAM-co-A) polymer, increased high temperature-responsiveness from PNIPAM and fluorescent properties from nucleobase (adenine), suggesting its potential as a theranostic system.

Combined pH/temperature sensitive nanogels were designed to facilitate controlled release at a specific tumor site with an acidic pH and to improve the thermal stability and biocompatibility of the materials. Nita L.E. et al. fabricated this type of system using poly (itaconic anhydride-co-3,9-divinyl-2,4,8,10-tetraoxaspiro (5.5) undecane) copolymer crosslinked with 1,12-dodecandiol [86]. Changing the polymer charge through a pH range (pH 4–11) can change the shrink-swell behavior of a nanogel, which affected drug release, were investigated by varying the comonomer ratio in the copolymer chain. Moreover, temperature responsiveness was also affected by the different comonomer ratios that caused the hydrodynamic radius and shrink-swell behavior of the nanogels to change. In addition, an interesting host-guest interaction between dextran grafted benzimidazole and thiol-β-CD was fabricated and used as a nanogel polymer chain [87]. An oxidative hydrosulfide group was employed as crosslinking group and GSH sensitive marker. Consequently, the size of the nanogels increased after being treated with GSH at pH 7.4, which can be inferred to be its response in the reductive cancer environment. The system was also designed to respond to acidic pH, therefore drug release from the nanogels increased at pH 5.3 without GSH. 

Lou S. et al. fabricated hepatocyte-specific multi-responsive nanogels consisting of poly (6-Ovinyladipoyl-D-galactose-ss-N-vinylcaprolactam-ss-methacrylic acid) or P(ODGal-VCL-MAA) [34]. β-D-galactose (GAL) or N-acetylgalactosamine that included asialogly co-protein receptor (ASGP-R) ligands, which are abundantly expressed in mammalian hepatocytes. The hepatocyte specificity of the ODGal ligands was identified using human hepatoma HepG2 cells. The cytotoxicity of the DOX-loaded P (ODGal-VCL-MAA) nanogels was significantly improved compared with those without ODGal ligands. The multi-responsiveness of the nanogels was observed in various temperature, pH, and reductase conditions. A higher phase transition temperature was observed at higher ODGal concentration. The nanogel’s pH-dependent drug release behavior was also investigated, the release rate of the loaded DOX increased as the pH decreased from 7.4 to 6.0. In the presence of GSH, increased drug release in the elevated reductase condition was observed. 

An effective delivery system for cancer therapy was demonstrated using crosslinked hyaluronic acid (HA) and keratin to fabricate multi-responsive nanogels [88]. Keratin is a hair protein that is biocompatible with no immunogenicity due to the abundant cysteines and sulfhydryl bonds in its structure that can be degraded by several enzymes, including trypsin. Additionally, keratin can increase the production of intracellular nitric oxide (NO) that attacks tumor cells, enhancing the cytotoxicity of chemotherapeutic substances. Furthermore, HA binding to CD44 promotes cell internalization. Due to HA’s negative charge, a large amount of positively charged DOX can occupy the nanogels surface. These well-designed nanogels displayed high drug encapsulation, combining pH-, trypsin-, and GSH-responsive drug release, as well as increased efficacy promoted by increased NO levels at the targeted cancer cells. 

Graphene oxide (GO), an inorganic compound, exhibits a distinct drug loading capacity. Due to its strong polarity, GO forms an electrostatic interaction with some cancer therapeutics, and it was combined with PNIPAM monomer to improve thermal responsiveness when exposed to NIR light, as well as enhancing its colloidal stability [89]. The results demonstrated the optimized DOX loading capacity together with improved colloidal stability and biocompatibility of the native GO. Furthermore, the multiple stimuli-response components provided targeted drug delivery under acidic and reducing conditions that were specific to the cancer cell microenvironment [90]. An in vitro cytotoxic study in HeLa cells indicated the local efficacy of the DOX-loaded nanogels after NIR light exposure. These nanogels demonstrated higher cellular uptake and bioimaging potential, confirming from the results of confocal laser scanning microscope analysis.

Moreover, GO-DOX coated with crosslinked hyaluronic acid nanogels were used as a theranostic system [91]. When incorporated with GO, the nanogels emitted light when exposed to red-light radiation that can be used as cancer-cell tracking. The loaded DOX can be specifically released at low pH and red light stimulation and the crosslinked hyaluronic acid specifically targets the CD44 receptors on A549 cells. The specificity of the nanogels decreases the side effects of the anti-cancer drugs and increases the accuracy of tumor/cancer identification. 

## 4. Biomedical Applications

As smart drug carriers, the modified polymeric nanogel systems are expected to enhance the efficiency of drug delivery, which can reduce side effects by enhancing the specificity to target sites and improve the drug’s pharmacokinetics that can prolong the circulation time, leading to a greater bioavailability and an elevated therapeutic efficacy. Moreover, with the stimuli-sensitiveness of polymers, polymeric nanogels can act as diagnostic tools for tracking specific organs or tissue with a higher precision. Furthermore, there are various modifications based on a combination of therapeutic and diagnosis, the so-called theranostic systems, which indicate the multiple functionalities of the modified nanogels.

### 4.1. Therapeutics 

To enhance the therapeutic properties of the nanogels, nano-carriers were designed to release their payloads upon the application of a stimulus, known as stimuli-responsive nanogels. The nanogels formulated as a therapeutic eye drop were typically made of chitosan, which was known for its mucoadhesive properties [92]. A chitosan derivative, N-succinyl-chitosan, was synthesized with self-crosslinking properties that stabilize the nanogel system. The results suggested the potential of this nanogel as a mucoadhesive enhancer due to the higher affinity to mucous epithelium compared with native chitosan. Additionally, the nanogels displayed a double-step release; an initial burst that results in a high therapeutic concentration in a short period, followed by a consequent sustained release to maintain the therapeutic level of the drug in a prolonged fashion.

The development of biopharmaceutical products containing macromolecules, i.e., proteins, has increased. The major limitation of protein delivery is maintaining its stability during storage or exposure to physiological enzymes, which affects its structure. Hemoglobin (Hb), a functional protein that transports oxygen, carbon dioxide, and other gases to support normal body functions in mammals, was used as a model protein in Wei X. et al. [93] Hb was loaded into a functionalized nanogel containing dextran grafted with succinic anhydride to promote self-assembly, and dopamine (DA) to modify the hydrophobicity of the nanoparticles. A gas-binding capacity study revealed that the nanogels demonstrated a positive trend in binding and releasing CO, N_2_, or O_2_ without the loss of Hb’s inherent bioactivities. Higher oxygen affinity compared with free Hb and increased stability of the protein in physiological buffers were observed. The nanoparticles also showed an enhanced biocompatibility and hemo-compatibility features because there were no signs of significant cell death or hemorrhagic abnormalities in the tested conditions. 

Dandan Li et al. [94] used reducing-sensitive nanogels loaded with ovalbumin (OVA) as a model antigen for vaccine applications. Methacrylated dextran and trimethyl aminoethyl methacrylate (TMAEMA)-based nanogels were conjugated with pyridyldisulfide groups to facilitate the response to a presence of a reducing agent. In the circulatory system or extracellular matrices, which are considered to be non-reducing conditions, the OVA antigen was entrapped in the nanoparticles, enhancing its stability against physiological enzymes. The OVA release from nanogels occurred as a response to the reductive urokinase-type plasminogen activator (uPA) environment after cell internalization. Therefore, the antigen-presenting property can be enhanced by this reducing-responsive nanogel, because dendritic cells, a potent antigen presenting cell, can exploit this stimuli-responsiveness to promote MHC class I cascades. 

Another intriguing example is multifunctional polymeric nanogels that were developed for improving the specificity of a rescue drug for stroke and decrease fatal side effects, including pulmonary edema and intracranial hemorrhage. PEG-conjugated glycol chitosan hollow nanogels were used to stimulate drug release after ultrasonic stimulation, which limited their specific target release and enhanced uPA thrombolysis activity and stability in circulatory system. The developed nanogels with ultrasonic stimulation showed a decreased risk of hemorrhage because the loaded uPA did not penetrate the blood-brain barrier [95,96]. In addition, PNIPAM core-acrylic acid shell nanogels were fabricated to deliver fibrin to enhance the efficacy of tissue-type plasminogen activator (tPA) for treating disseminated intravascular coagulation [97]. The findings indicated that the dual functionalities of the nanogels, the thrombolytic effect on microthrombi that can reduce risk of organ failure and the ability to ameliorate blood clots at the injured sites, provide a promising tool for targeted tPA treatment together with a restoration the hemostatic balance in patients.

The tumor microenvironment displays distinctive conditions that allows for wide functional design of drug delivery systems. A co-release system of DOX and cisplatin made of a tri-coblock polymer, poly (acrylic acid-b-PNIPAM-b-acrylic acid), or PNA, was fabricated [98]. DOX was loaded into the core and electrostatically bound to the carboxyl groups of PNA, and cisplatin was incorporated into the shell. Due to the different loading compartments of the drugs, the DOX and cisplatin exhibited different release behaviors, resulting in synergistic tumor suppression. The DOX and cisplatin ratio can be optimized to achieve different release patterns. Moreover, the thermo-responsiveness of PNIPAM contributed to the sol-gel transition of the nanogels that was beneficial for targeted release at the tumor site.

Topical nanogel formulations have also been widely investigated. Zhu J. et al., fabricated chlorhexidine diacetate-loaded nanogels enclosed in aminoethyl methacrylate hyaluronic acid and methacrylated methoxy polyethylene glycol crosslink system, referred to as Gel@CLN hydrogels as a wound dressing. The results demonstrated that Gel@CLN hydrogels reduced infection by *E. coli* and *S. aureus* with prolonged inhibition for up to 10 days. Gel@CLN hydrogels treatment also accelerated wound healing due to its high water content, which enhanced its moisturizing effects and supported rapid wound closure [99]. 

### 4.2. Diagnotics and Theranostics

Apart from using nanogels as drug delivery systems to improve their therapeutic effects, the nanogels also possess multi-functional features that can be used for diagnosis. QD-containing polymeric nanogels that emitted a specific light/fluorescent signal were used as biological dyes for live cells or in vivo imaging. Multi-responsive biosensing nanoparticles containing Cadmium-Selenium (CdSe) QDs shielded with chitosan-poly (methacrylic acid) were fabricated to combine the optical properties of QDs and the pH-responsiveness of the modified chitosan [82]. Therefore, the controlled release of loaded drugs occurred at a pH of 5.0–7.4, and the internalization of the nanogels can be visually observed due to the optical activities of CdSe QDs. Thermo-responsive copolymers were used as a diagnostic device for high contrast MRI, by Kolouchova K. et al. [67]. Two block co-polymers, PHPMA or PMeOx, and a thermo-responsive PDFEA, were fabricated, and the materials self-assembled into nanoparticles at human body temperature. The nanogels were then used to encapsulate fluorine atoms as a probe for high sensitivity MRI. 

Various targeted diagnostic nanogel platforms have been evaluated for their potential biomedical application; however, for those containing metals, their toxicity is a concern. Hence, several core-shell structured nanogels were used to reduce the cytotoxicity by shielding the metals from being exposed to the cell surface. Gold nanoparticles (AuNPs)-based chitin-MnO_2_ ternary composite nanogels (ACM-TNGs) were fabricated as a radio-assisted cancer therapy [100]. This system was designed to reduce the toxicity of MnO_2_ nanorods that absorbed low frequency radio waves. To minimize the toxicity from the metals, chitin nanogels were used as a biocompatible shell that wrapped the AuNPs and MnO_2_ nanorods. 

A cytotoxicity study using L929 HDF, MG63, T47D, and A375 cell lines demonstrated the cytocompatibility of ACM-TNGs and that their cell internalization did not alter cell morphology. Moreover, this nano-system also killed breast cancer cells at a 100 watts/2 min radio frequency. The potential of the nanogel as a cell tracker was investigated using NIR light-triggered drug release [101]. 

Kimura A. et al. used ultra-small Galodium-gelatin nanogels as an MRI contrast agent because the coated gelatin shell minimizes the risk of nanoparticles leaking through the blood-brain barrier and blood-cerebrospinal fluid barrier [102]. The developed nanogels provided high contrast MRI images with low toxicity to cultured cells. The in vivo biodistribution in tumor-bearing mice demonstrated rapid renal clearance with no accumulation in the kidney and liver, including over long-term observation. Moreover, no nanoparticles were found in the blood stream and cerebrospinal fluid, which confirmed the high safety profile of the developed nanoparticles as a MRI contrast agent.

Theranostic is a term used to describe tools, devices, or systems that have combined diagnostic and therapeutic features. For a drug delivery system, these platforms were designed to improve the therapeutic potential and decrease side effects, along with being able to diagnose or indicate the drug’s biodistribution after administration. Wu W. et al. used pH-responsive polymers, PNIPAM-co-acrylic acid with embedded Au nanoparticles, as an imaging probe [103]. The nanogels exhibited pH-sensitive behavior due to the protonation of their acrylic acid moieties, and increased hydrophobic drug loading capacity from the temperature-sensitive hydrophobic NIPAM groups. In another study, folate-terminated poly (ethylene glycol)-modified hyaluronic acid crosslinked with carbon dots was synthesized to target tumor cells that highly express folate receptors [90]. This system provided pH- and acidic-sensitive drug release that should be specific to tumor tissue conditions, and the carbon dots could be used for bioimaging.

A graphene-DOX-entrapped hyaluronic acid nanogel was evaluated as a theranostic system [91]. The nanogels emitted light from the entrapped graphene when exposed to red-light radiation, which is advantageous for cancer-cell imaging. Due to the high expression of CD44, hyaluronic acid receptors, DOX-targeted release via pH/red light-responsiveness was seen in cultured non-small cell lung cancer A549 cells. The specificity of the nanogels decreased the side effects of the cancer drug and increased the accuracy of the cancer identification. 

Peng N. et al. formulated novel hybrid nanogels composed of a disulfide-modified alginate shield covering superparamagnetic iron oxide nanoparticles (SPIONs, an MRI probe, for theranostic applications [104]. The nanogels can be used as an imaging agent in acidic and reductive environments, which was beneficial for tumor-targeted release and diagnostic targetability. The combination of SPIONs and biocompatible alginate derivative as a delivery and cell imaging system also exhibited high DOX loading and high toxicity to tumor cells. 

In conclusion, the biomedical uses of nanogels require the biocompatibility and specific designs of the nanogels to minimize the side effects to surrounding tissue and enhance the efficacy and stability of active drugs, respectively. The core-shell structure of the nanogels protects the encapsulated drugs and increases the specificity to the nanocarriers because its components influence the responsiveness to various stimuli (physiological and external environment). In addition, the adjustable design of polymers results in the stability of active compounds in the physiological environment and having feature-specific functions. Moreover, the stimuli-responsive behavior of nanogels allows them to be used as biocompatible diagnostic tools that provide precise results. The practical biomedical use of nanogels is being increasingly investigated to develop efficient and specific medical tools and advance the use of nanotechnology in drug delivery systems. 

## Data Availability

Not applicable.
